# Optogenetic Dissection of Temporal Dynamics of Amygdala-Striatal Interplay during Risk/Reward Decision Making

**DOI:** 10.1523/ENEURO.0422-18.2018

**Published:** 2018-12-10

**Authors:** Debra A. Bercovici, Oren Princz-Lebel, Maric T. Tse, David E. Moorman, Stan B. Floresco

**Affiliations:** 1 Department of Psychology and Djavad Mowafaghian Centre for Brain Health, University of British Columbia, Vancouver, BC, CANADA, V6T 1Z4; 2 Department of Psychological and Brain Sciences, Neuroscience and Behavior Program, University of Massachusetts, Amherst, MA, USA, 01003

**Keywords:** accumbens, amygdala, decision making, optogenetic inhibition, reward

## Abstract

Decision making often requires weighing costs and benefits of different options that vary in terms of reward magnitude and uncertainty. Previous studies using pharmacological inactivations have shown that the basolateral amygdala (BLA) to nucleus accumbens (NAc) pathway promotes choice towards larger/riskier rewards. Neural activity in BLA and NAc shows distinct, phasic changes in firing prior to choice and following action outcomes, yet, how these temporally-discrete patterns of activity within BLA→NAc circuitry influence choice is unclear. We assessed how optogenetic silencing of BLA terminals in the NAc altered action selection during probabilistic decision making. Rats received intra-BLA infusions of viruses encoding the inhibitory opsin eArchT and were well trained on a probabilistic discounting task, where they chose between smaller/certain rewards and larger rewards delivered in a probabilistic manner, with the odds of obtaining the larger reward changing over a session (50–12.5%). During testing, activity of BLA→NAc inputs were suppressed with 4- to 7-s pulses of light delivered via optic fibers into the NAc during discrete task events: prior to choice or after choice outcomes. Inhibition prior to choice reduced selection of the preferred option, suggesting that during deliberation, BLA→NAc activity biases choice towards preferred rewards. Inhibition during reward omissions increased risky choice during the low-probability block, indicating that activity after non-rewarded actions serves to modify subsequent choice. In contrast, silencing during rewarded outcomes did not reliably affect choice. These data demonstrate how patterns of activity in BLA→NAc circuitry convey different types of information that guide action selection in situations involving reward uncertainty.

## Significance Statement

Amygdala projections to the nucleus accumbens (NAc) form a neural circuit that has been implicated in guiding risk-related decision making, and dysfunction in this circuitry is thought to underlie aberrant decision making observed in a variety of psychiatric illnesses. The present study used temporally-discrete optogenetic inhibition to identify how activity in amygdala inputs to the accumbens, during different phases of the decision process, promotes optimal decision making. These findings provide novel insight into the mechanisms through which these circuits contribute to normal and abnormal decisions involving reward uncertainty.

## Introduction

Optimal reward-seeking often requires evaluations of the relative costs and benefits associated with different actions. Among the various costs that may be associated with different rewards (Winstanley and Floresco, 2016), decisions involving uncertainty often require integration of information pertaining to reward magnitudes and action/outcome history to infer the probability of obtaining different rewards and direct choice toward options that may be more profitable.

Studies in humans and animals have implicated various nodes within cortical, limbic and striatal circuitry in mediating different aspects of risk/reward decision making (Orsini et al., 2015a). Among these, the nucleus accumbens (NAc) has been proposed to be a focal point where information processed by prefrontal and limbic regions pertaining to reward magnitude, history, and subjective value are integrated to bias actions toward more preferred rewards (Mogenson et al., 1980; Kuhnen and Knutson, 2005; Nicola, 2007; Floresco, 2015). The basolateral amygdala (BLA) provides key input to the NAc (Brog et al., 1993; Britt et al., 2012) and has been implicated in assigning value to reward options and promoting actions toward those of greater subjective value (Balleine and Killcross, 2006; Zeeb and Winstanley, 2011; Orsini et al., 2015b; Averbeck and Costa, 2017). In this regard, previous work using a probabilistic discounting task in rodents have shown that the BLA→NAc pathway forms a functional circuit that biases choice toward larger, more preferred rewards. Lesions/inactivation of either nucleus, or functional disconnection of these regions reduces preference for larger, uncertain rewards relative to smaller, certain ones, most prominently when animals show biases toward large/risky options (Cardinal and Howes, 2005; Ghods-Sharifi et al., 2009; Stopper and Floresco, 2011; St Onge et al., 2012).

Risk/reward decision making is a dynamic process that may be partitioned into different phases, including deliberation before action selection and subsequent evaluation of action outcomes that may influence subsequent choice biases. Neurophysiological studies suggest that these different phases of the decision process are likely mediated by temporally discrete patterns of activity within different nodes of cortico-limbic-striatal circuitry. For example, Sugam et al. (2014) recorded activity from NAc neurons in rats choosing between small/certain and larger, uncertain rewards. They reported that a subpopulation of NAc neurons exhibited phasic changes in firing before action selection, with this activity being more robust when animals chose their more preferred option. In addition, some NAc cells fired in response to reward delivery, whereas another population increased firing following non-rewarded choices. In a similar vein, BLA neurons display greater increases in phasic activity when presented with a discriminative stimulus signaling reward availability compared to that evoked by a non-rewarded stimulus, and these BLA responses drive similar patterns of activity in the NAc (Ambroggi et al., 2008). Moreover, some BLA neurons show increased activity in response to unexpected declines in reward value (Roesch et al., 2010). These latter findings suggest that differential patterns of activity in NAc neurons that occur during different phases of the decision process may be driven in part by inputs from the BLA. However, it remains unclear how activity in these circuits occurring before choices or after their outcome are realized, contribute to action selection during decision making.

The present study sought to address this issue by using temporally-specific optogenetic inhibition of BLA projection terminals in the NAc, to ascertain how suppressing activity in this pathway during different task events may alter risk/reward decision making. Rats received intra-BLA infusions of an adeno-associated virus (AAV) encoding for the inhibitory opsin eArchT and were well trained on a probabilistic discounting task where they chose between small/certain and large/risky rewards. During testing, light was delivered into the NAc to suppress input from the BLA to this nucleus during distinct phases of the decision process. In a separate experiment, we confirmed the effectiveness of this procedure in suppressing BLA-evoked firing of NAc neurons, using *in vivo* electrophysiological recordings.

## Materials and Methods

### Subjects

Male, Long Evans rats (Charles River Laboratories) weighing ∼275–300 g on arrival were group housed and provided food ad libitum for one week. Following daily handling and acclimatization to the colony, rats underwent stereotaxic viral infusion surgery into the BLA and were subsequently single housed for the remainder of the experiment. Rats were food restricted to ∼85% of their free-feeding weight before beginning operant behavioral training. Their weights were monitored daily and food was adjusted to maintain a weight gain of ∼5 g per week. All animal procedures were performed in accordance with the University of British Columbia animal care committee’s regulations.

### Stereotaxic surgery

Forty-eight hours before surgery, rats were given food ad libitum. Rats were given a subanesthetic dose of ketamine (50 mg/kg) and xylazine (5 mg/kg) intraperitoneally and maintained on isoflurane (5%) for the procedure. They were placed into a stereotaxic frame and secured with earbars (flat skull) and analgesia was administered subcutaneously (Anafen, 10 mg/kg). Over the course of this experiment, rats received two surgeries, the first involving infusions of virus into the BLA before behavioral training, and the second entailing implantation of optical fibers into the NAc after initial training. During these surgeries, burr holes were drilled into the skull above the BLA or NAc. Over the course of these experiments, animals were subjected to two surgical procedures at different time points.

#### Viral infusion

Before any behavioral training, separate groups of rats received 1.0-µl infusions of a solution containing a virus encoding the inhibitory opsin rAAV5-CaMKIIα-eArchT3.0-eYFP (to selectively promote protein expression in glutamatergic neurons) or rAAV5-CaMKIIα-eYFP as a control vector (University of North Carolina Vector Core). Infusions were made bilaterally into the BLA via microinfusion pumps (coordinates from bregma: –3.2 mm anteroposterior; ±5.1 mm mediolateral; –7.6 mm dorsoventral from dura) at a flow rate of 0.1 µl/min. Injectors were left in place for an additional 10 min following infusion to allow for virus diffusion in tissue. Incisions were sutured, and rats were given approximately two weeks to recover and then commenced the initial behavioral training, which lasted approximately five weeks.

#### Fiber optic implantation

After completion of their initial behavioral training, rats received their second surgery. Here, optic fibers consisting of 400-µm cores (Thorlabs) threaded through 2.5 mm-wide metal ferrules (Precision Fiber Products) were implanted over the NAc at a 12° angle (coordinates from bregma: +1.5 mm anteroposterior; ±1.5 mm mediolateral; –6.7 mm dorsoventral from dura). Ferrules were secured to the skull using four screws and dental cement to secure the assembly, leaving about half of the ferrules uncovered. Rats were permitted to recover from this surgery for one week before retraining and optogenetic testing.

### Apparatus

Behavioral testing was conducted in operant conditioning chambers (30.5 × 24 × 21 cm; Med-Associates) enclosed in sound-attenuating boxes. Each box was equipped with a fan with the purpose of providing ventilation and limiting extraneous noise. The chamber was fitted with a central food receptacle where sucrose food reward pellets (45 mg; Bioserv) were dispensed. Two retractable levers were located on either side of the food receptacle. The chamber was illuminated by a 100-mA house light located on the top center of the box opposite the food receptacle. Four infrared photobeams located just above the grid floors monitored locomotor activity (assessed by number of beam breaks). All data were recorded on a personal computer connected to the operant conditioning chambers. Lasers were controlled by Med PC hardware and software, which delivered a TTL+ pulse to lasers to initiate light delivery and a TTL– pulse to terminate it.

### Lever press training

After ∼5 d of food restriction, rats were given ∼30 pellets in their cage on the day before beginning operant training. On the first day of training, two pellets were placed in the food receptacle, the right or left lever was extended, and crushed reward pellets sprinkled on the extended lever. Animals were trained to lever press for pellets under a fixed ratio-1 schedule until a criterion of 60 presses in 30 min for one lever, and on the next day, the other lever. Rats were subsequently trained on a simplified version of the full task, consisting of 90 trials. On each trial, rats were presented with one of the levers that, if pressed within 10 s, would deliver one pellet with a 50% probability. If the lever was not pressed within this time, it was retracted and the trial was scored as an omission. Trials occurred every 40 s. Rats trained until a criterion of <10 omissions for a minimum of two consecutive days (∼4 d of training). During the next phase of training, rats learned to choose between one lever associated with a larger, four-pellet reward (delivered with a 50% probability) and another lever that always delivered a one-pellet reward. Assignment of the large-reward lever was counterbalanced across animals. Sessions consisted of 72 trials portioned into four blocks of 18 trials. The first eight trials of each block were forced choice, where only one lever was inserted (randomized in pairs), and the remaining 10 trials were free choice, where both levers were inserted. Rats were trained until they chose the large reward lever on >60% of the free choices (∼3 d).

### Probabilistic discounting training

The task was similar to that described by Stopper et al. (2014) that was used to investigate how temporally-discrete manipulations of phasic dopamine activity alters risk/reward decision making. Each 40-min daily session consisted of 60 trials separated into two, 30-trial blocks (10 forced- followed by 20 free-choice trials). Rats were trained 5–7 d per week. For each rat, one lever was designated the small/certain lever and the other lever was designated the large/risky lever (same as the last phase of training). Trials commenced every 40 s and began with illumination of the house light and 4 s later, one (forced choice) or both (free choice) levers were inserted into the chamber. Rats were given 10 s to press a lever otherwise the lever(s) retracted and the trial scored as an omission. Selection of either lever caused both to retract. Choice of the small/certain option always delivered one pellet. Choice of the large/risky option delivered four pellets at changing probabilities. For the first 30 trials, the probability of reward delivery was set at 50% (making it a more advantageous selection vs the small/certain option). For the last 30 trials, the reward probability was 12.5%, so that the small/certain option had greater utility versus the large/risky one. On trials where a choice was rewarded, the house light remained illuminated for another 3 s, whereas after non-rewarded choices or omissions, the light was extinguished coincidentally with lever retraction. Pellet delivery was initiated immediately after a lever press, and multiple pellets were delivered 0.5 s apart.

Rats were trained until the group demonstrated stable choice behavior (∼30 d), evaluated by analyzing data from three consecutive days using a two-way repeated measures ANOVA, with day and trial block as the two factors. Behavior was deemed stable when there was no main effect of day and no day × block interaction (at *p* > 0.10). Once stable patterns of choice were displayed, rats were given food ad libitum for 3 d before undergoing fiber optic implantation, as described in the preceding section. Following recovery from surgery, rats were retrained on the task. During retraining, ferrules were connected to dummy fiber optic patch cables, encased in stainless-steel spring coils and tethered to a rotary joint that permitted free movement through the chamber. This procedure habituated animals to the procedures they would experience during testing. Once stable behavior was re-established (∼15 d), optogenetic test sessions commenced.

It is important to note that despite the extended training rats received on this task, rats continued to display prominent discounting of the large/risky option as reward probabilities decreased across blocks, indicative that they remained sensitive to changes reinforcer probability. Furthermore, previous studies have shown that choice behavior during these types of tasks remains sensitive to reinforcer devaluation (i.e., prefeeding) even after many weeks of training (St Onge and Floresco, 2009; Stopper and Floresco, 2014). Thus, although animals had extensive experience with this task, it is likely that their choices were guided by representations of action-outcome contingencies and were monitoring risks and reward values to optimize outcomes in a goal-directed manner, rather than using a habitual strategy.

### Optogenetic inhibition

**Figure 1.**
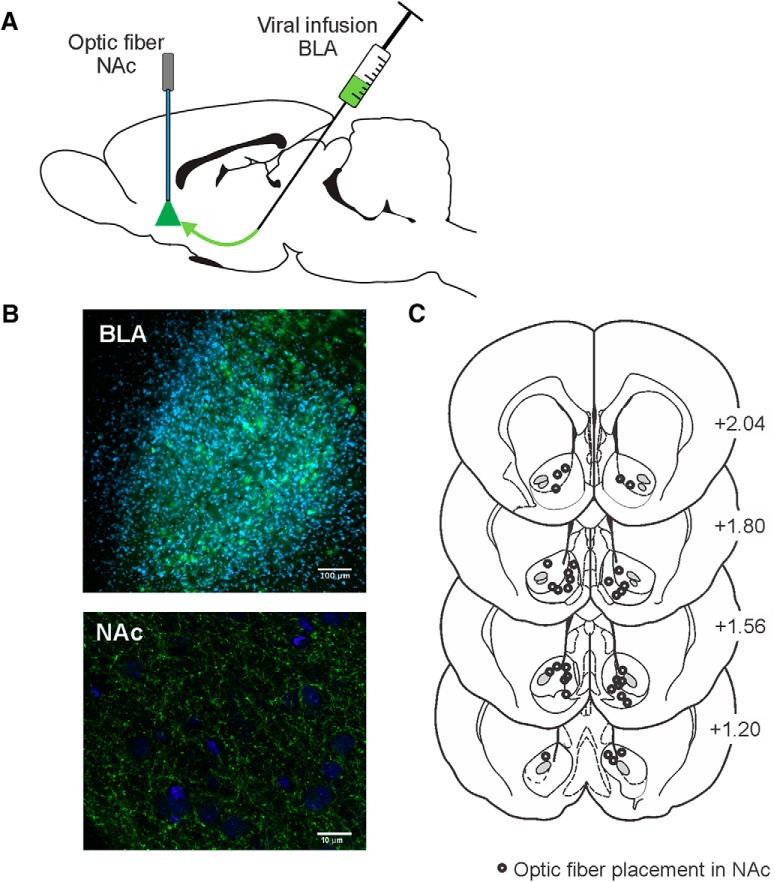
Optogenetic design, histology, and fiber placements for the behavioral experiments. ***A***, Viral infusions were made into BLA, and laser light via optic fibers was delivered to NAc to suppress activity in BLA terminal inputs. ***B***, top panel, Representative slice at 10× of BLA expression within the BLA (blue is DAPI; green is eYFP). Bottom panel, Representative slice at 63× of BLA terminal expression in NAc (blue is DAPI; green is GFP). ***C***, Location of fiber optic placements in NAc. Numbers correspond to mm from bregma.

Testing commenced approximately two months post viral infusion. This time frame permitted the opsins that were initially expressed in the BLA to be trafficked down axons and expressed in terminals in the NAc where they could be activated by laser light (Fig. 1*A*,*B*). Before a test session, the indwelling ferrules were tethered to 200-µm core optic fiber patch cables (Thorlabs) connected to a dual-channel optical rotary joint (Doric Lenses) that split laser light so that each channel emitted 50% of the light intensity output. The rotary joint was attached to an optic fiber patch cable (Thorlabs) that was connected to green (532 nm) diode-pumped solid-state lasers (Laserglow Technologies). On test days, the intensity of the laser was measured with a light meter (Thorlabs) so that ∼30 mW of 532-nm light was delivered per split fiber.

Each optogenetic test consisted of a 3-d sequence similar to that used in other studies using temporally-discrete manipulations of neural activity during different phases of decision making (Stopper et al., 2014; Orsini et al., 2017). The first 2 d of the sequence were baseline days, where a rat was connected to the fiber optic cables, but no light was delivered. On the subsequent test day, animals were connected to the lasers and received brief pulses of laser light to suppress activity of BLA terminals in the NAc during discrete task events. Most rats received two test sequences for each type of optogenetic manipulation, separated by at least 3 d of retraining. Behavioral data from the baseline days were averaged and compared to those obtained on the laser test days. Across all of the different types of tests, there were no differences in choice behavior across the four baseline days (all *F*s < 3.50, n.s.). Animals completed all tests for a particular manipulation before moving on to the next type of manipulation. The order with which the different types of tests were administered was counterbalanced across rats. Some experiments had considerable attrition rates as a result of damage to headcaps. Ultimately, some rats did not receive all silencing tests, resulting in a differing number of subjects in each analysis. Two rats displayed abnormal discounting behavior (i.e., they showed a greater bias for the large/risky lever in the 12.5% block vs the 50% block) and their data were not included in the final analyses. Rats infused with the control virus not containing the inhibitory opsin eArchT were only tested on manipulations that induced significant changes in choice in the eArchT virus group.

#### Pre-choice inhibition

In one experiment, BLA inputs to the NAc were suppressed before animals made a choice. Laser light was initiated 4 s before lever extension (coinciding with illumination of the house light signaling the start of a trial) and terminated either when a choice was made or 10 s elapsed after lever extension (omission). Under these conditions, light was delivered for 4–10 s each trial depending on response latency (on average ∼5 s). Previous studies have shown that when rats choose between risky and certain rewards, NAc neural firing begins to increase during a deliberation period a few seconds prior to initiation of a choice (Sugam et al., 2014). As such, we chose to inhibit BLA→NAc a few seconds before lever insertion to maximize the inhibition of activity during this period. In this experiment, laser light was delivered only during free-choice trials, as we were primarily interested in how activity in this pathway influenced choice of the two options.

In separate series of experiments, we inhibited BLA inputs to the NAc after different outcomes of choices. In all of these experiments, laser light was delivered during the outcome of interest on both forced and free-choice trials, since regardless of trial type, information about probability gathered from these outcomes influenced subsequent choice biases.

#### Risky “loss” inhibition

In another experiment, we inhibited BLA inputs to the NAc only on trials where animals chose the risky option and did not receive a reward (“risky loss”). Here, lasers were illuminated on all free- and forced-choice trials when a rat selected the large/risky lever and did not receive the larger reward. Laser light was initiated immediately after these choices and was terminated 7 s post-lever press.

#### Risky “win” inhibition

Another experiment inhibited activity in the BLA→NAc pathway after rewarded risky choices (“risky wins”). On these test days, light was delivered on all free- and forced-choice trials after a rat selected the large/risky lever and received the larger reward. Lasers were left on for the 7 s post lever press, which would have overlapped with pellet delivery and consumption.

#### Small/certain win inhibition

This experiment inhibited activity in BLA→NAc pathway after a small/certain choice (“small wins”). During these tests, lasers were illuminated on for all free- and forced-choice trials immediately after a rat selected the small/certain lever. Lasers were left on for the 7 s post lever press, which included the time it took for pellet delivery and consumption.

#### Intertrial interval (ITI) inhibition

To ascertain that the effects of outcome-associated silencing of the BLA→NAc pathway was attributable to inhibiting neural activity that was temporally linked to these events, a control experiment tested the effects of inhibiting this pathway for 7 s during the 40-s ITI. For all free- and forced- choice trials, light pulses were delivered during the ITI, on a random interval starting 6–14 s after the start of the ITI (i.e., following a lever press or omission).

### *In vivo* single unit recordings

A separate cohort of rats underwent viral infusion surgery and were allowed to recover for approximately two months before being used for electrophysiological experiments. Some rats received infusions of the virus encoding for eArchT3.0-eYFP, and others with a control virus that only encoded for eYFP. All rats were given food ad libitum post-viral infusion before physiologic recordings. Rats were anesthetized with urethane (1.5 mg/kg) and inserted into a stereotaxic frame. The rat’s scalp was incised, and burr holes were drilled in the skull at coordinate above the BLA and the NAc; stimulating and recording optical microelectrodes (optrodes) were lowered at the coordinates described in the subsequent section.

The electrophysiological cell-searching and recording procedures were adapted from Floresco et al. (2001) and Floresco and Tse (2007). Recording microelectrodes were constructed from 2.0-mm outer diameter borosilicate glass capillary tubing (World Precision Instruments) using a vertical micropipette puller (Narishige). The tips of the electrodes were broken back against a glass rod to ∼1-µm tip diameter. Optrodes were constructed by coupling the microelectrode to a stripped end of a 200-µm core patch cable which was connected to a 532-nm solid-state laser, so light could be emitted adjacent to the tip of the recording electrode. The signal from the glass microelectrode was amplified and filtered (500–2000 Hz) using an X-Cell3+ microelectrode amplifier (Frederick Haer Company). Action potential data were acquired, discriminated from noise, stored, and analyzed using Spike 2 software (Cambridge Electronics Design) running on an Intel-based personal computer with a data acquisition board interface (micro 1401 mk II; Cambridge Electronics Design).

A stimulating electrode connected to an Iso-Flex optically-isolated stimulator (AMPI) that received programmed pulses from a Master-8 pulse generator (AMPI) was lowered into the BLA (coordinates from bregma: –3.0 mm anteroposterior; ±5.1 mm mediolateral; –7.2 mm dorsoventral from dura). Afterward, a cell-searching procedure began, wherein the optrode was lowered into the NAc with a hydraulic microdrive (coordinates from bregma: +1.4 mm anteroposterior; ±1.2 mm mediolateral; ranges between 5.0–8.0 mm ventral from dura), while single-pulse electrical stimulation was delivered to the BLA every 8 s. Searching continued until a NAc neuron that exhibited a reliable, monosynaptic action potential in response to BLA stimulation was isolated. Stimulation currents were then titrated to evoke a baseline firing probability of ∼50% (for eArchT3.0 group, average current: 1217 ± 159 µA, current range: 400–2000 µA; for eYFP group, average current: 1290 ± 297 µA, current range: 300–2000 µA). Once a stable baseline was established (40 sweeps over ∼5 min), a second set of stimulations were administered. Here, the laser was turned on for 4 s (with 5 or 10 mW power), starting 2 s before electrical stimulation of the BLA (40 sweeps). This was to confirm whether local light application could suppress BLA-evoked firing of NAc neurons. Immediately after test sweeps, another set of 40 sweeps were tested without laser delivery, to assess recovery of evoked neural firing. We typically obtained between one to three cells per rat tested. At the end of data collection, rats were killed via transcardial perfusion and brains were obtained for histologic analysis.

### Histology

Rats were killed via transcardial perfusion with 4% paraformaldehyde. Brains were fixed in 4% paraformaldehyde for 24 h and then stored in PBS with sodium azide. Each brain was sliced in 50-μm sections using a vibratome (Leica). NAc sections were treated with citric acid at 95°C for 10 min, incubated for 3 d in PBS with 10% Triton X-100, 3% horse serum and chicken anti-GFP (1:500; GFP1020, Aves Labs). Visualization was performed with anti-chicken 488 secondary antibody (Jackson Laboratory) diluted 1:250 in PBS with 10% Triton X-100, 3% horse serum for 60 min at room temperature. BLA and NAc sections were mounted onto slides, counterstained, and coverslipped using Fluoromount-G with DAPI (eBioscience). Viral expression was verified in the BLA using a 10× objective and terminal expression in the NAc was localized using a 63× objective (Fig. 1*B*). Placements of fiber implants were localized on a confocal microscope (Leica SP8) using a 20× water-immersion lens (Fig. 1*C*) referencing a neuroanatomical atlas (Paxinos and Watson, 2005).

### Data analysis

#### Probabilistic discounting

For each behavioral manipulation, the primary dependent variable was the proportion of choices of the large/risky option, controlling for trial omissions. This was calculated by dividing the number of large/risky choices made in a block by the total number of choices made in that block. Choice data from the two probability blocks were further partitioned into sub-blocks of 10 trials to assess whether inhibition altered discounting within a probability block. These data were analyzed with three-way repeated measures ANOVAs, with treatment (optogenetic inhibition vs baseline), probability block (50% and 12.5%) and sub-block as three within-subjects factors. For these experiments, the main effect of probability block was always significant (*p* < 0.001) and will not be mentioned further. Likewise, the main effect of sub-block was also significant (*p* < 0.05), indicating that within each probability block, rats tended to choose the risky option less as the block progressed. In these analyses, a treatment × probability block interaction indicates that optogenetic inhibition differentially altered choice over the 20 trials within each probability block relative to baseline. Moreover, a treatment × probability block interaction, combined with a lack of a three-way interaction would indicate that the effects of inhibition on choice within a particularly block did not differ significantly over the first versus last 10 trials of that block relative to baseline. For these ANOVAs, sphericity was not a concern, as all factors were comprised of only two levels. Additionally, while repeated measures ANOVAs are robust to violations of the assumption of normality, checks for normality were run and the distributions did not deviate from normal (all *p*s >0.20).

If there was a significant effect of optogenetic inhibition on choice behavior, additional choice-by-choice analyses evaluated whether these changes were associated with changes in reward sensitivity (win-stay behavior) and negative feedback sensitivity (lose-shift behavior). The analyses compared each free-choice to the outcome of a previous choice of the risky lever. A win-stay ratio was calculated based on the proportion of times rats chose the risky lever following previous receipt of the large reward (a risky win) over the total number of large rewards obtained, and was used as an index of reward sensitivity. The lose-shift ratio was calculated based on the proportion of times rats chose the safe lever following previous non-rewarded choice (a risky loss) over the total number of non-rewarded choices, and was used as an index of negative feedback sensitivity. Both these values were analyzed together in a two-way ANOVA with treatment and feedback sensitivity (win-stay/lose-shift) as the two within-subject factors. For all multi-factor ANOVAs, when a significant statistical interaction was obtained, simple main effects analyses were conducted using one-way ANOVAs where appropriate. Additionally, the total number of trial omissions, response latencies (the time between lever insertion and lever press), and locomotion were analyzed with one-way repeated measures ANOVAs.

#### *In vivo* single-unit recordings

Evoked-firing probabilities were calculated by dividing the number of action potentials observed by the number of stimuli administered. Changes in spike probabilities were used as an index of the effect of optogenetic inhibition on the magnitude of change in NAc neuronal activity produced by subsequent BLA stimulation. This was analyzed with one-way ANOVAs with phase (baseline vs laser application) as the within-subjects factor. Only cells that showed evoked responses during the post-laser recovery phase were included in the analysis to ensure that reductions in firing were not attributable to electrode drift.

## Results

### Histology

Confirmation of eYFP fluorescent protein in the BLA revealed robust expression in cell bodies encompassing the entire anterior–posterior range (Fig. 1*B*, top). Terminal YFP expression in the NAc was scattered within both the core and shell subregions and visual inspection showed terminals in close proximity to NAc neurons (Fig. 1*B*, bottom). Fiber optic placements ranged from +1.20 to +2.04 mm anterior-posterior to bregma and clustered observed primarily around the border region between the NAc core and shell subregions (Fig. 1*C*)

### Inhibition of BLA→NAc inputs before choice

**Figure 2.**
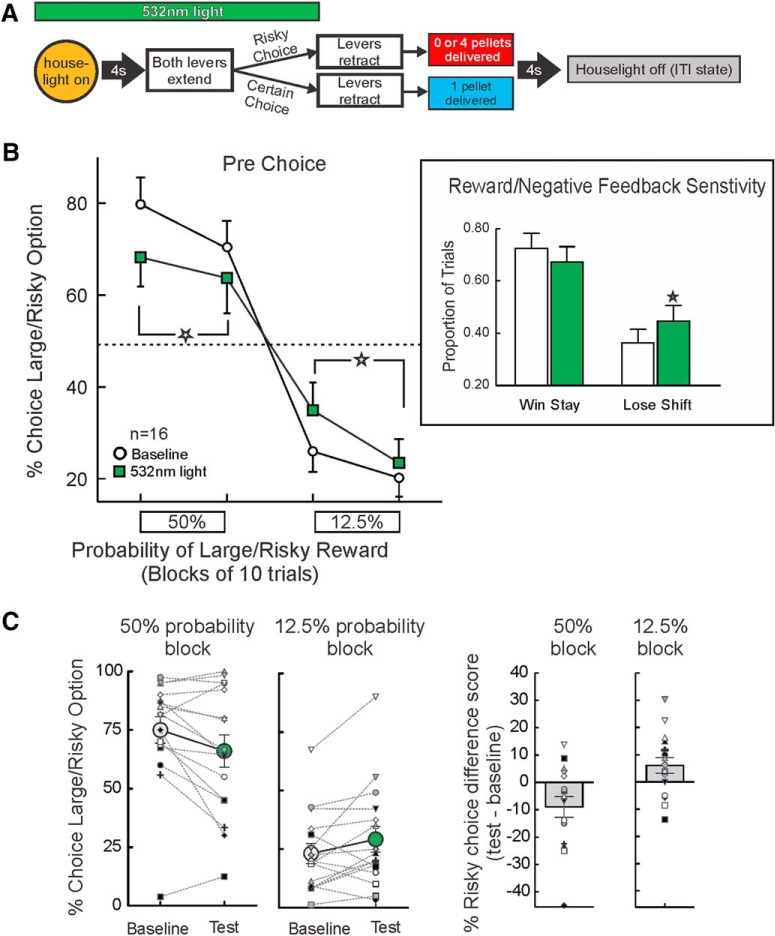
Inhibition of BLA terminals in the NAc before choice alters action selection during probabilistic discounting. ***A***, Optogenetic manipulation and task design. Light was delivered at the start of every free choice trial until lever press. ***B***, Percentage choice of the large/risky option under baseline conditions and during optogenetic tests. Inhibition before choice reduced risky choice during the higher, 50% probability block, and increased risky choice in the lower, 12.5% block (*n* = 16). Inset, Pre-choice optogenetic inhibition increased lose-shift behavior, but did not affect win-stay behavior. ***C***, Individual data, plotted in terms of the percentage of risky choices under baseline and optogenetic test conditions (left panels) and the difference score of these values between baseline and test (right panels). Large circles and bars display the group mean ± SEM for the left and right panels, respectively. In the majority of animals tested, optogenetic inhibition reduced/increased risky choice relative to baseline in the 50%/12.5% probability blocks, respectively. For this and all other figures, error bars are SEM, and stars denote *p* < 0.05 compared to baseline.

In 16 rats, we tested the effects of optogenetic inhibition of BLA terminals in the NAc before all free-choice trials (Fig. 2*A*). Under baseline conditions, rats displayed near-optimal patterns of choice, displaying a prominent bias for the large/risky option when this option had greater utility during the 50% block. During the 12.5% block, when the small/certain option would have yielded more reward, choice was biased away from this option. Suppressing BLA input during the deliberation period markedly altered choice relative to baseline. Analysis of these data revealed a significant treatment × probability block interaction (*F*_(1,15)_ = 16.44, *p <* 0.001; Fig. 2*B*) but no main effect of treatment (*F*_(1,15)_ = 0.27, *p* = 0.38). The three-way interaction was not significant (*F*_(1,15)_ = 2.08, *p* = 0.17). Simple main effects analyses on the treatment × probability block interaction revealed this was driven by a significant (*p* < 0.05) reduction in risky choice across the 20 trials of the 50% block on test days. In contrast, in the 12.5% block, optogenetic inhibition increased in risky choice compared to baseline.

Figure 2*C* displays the individual risky choice data for this experiment, averaged across the 20 trials of each probability block. These are plotted in terms of the percentage of risky choices under baseline and optogenetic test conditions (left panels) and the difference score of these values between baseline and test (right panels). As displayed in the left panels, there was considerable variability across rats in terms of the percentage of risky choices. However, the significant treatment × probability block interaction is reflected by the fact that optogenetic inhibition reduced risky choice relative to baseline values in the majority (11/16) rats tested in the 50% block. Conversely, in the 12.5% block, 11/16 rats increased risky choice relative to baseline.

To further dissect whether these changes in risky choice were related to alterations in sensitivity to preceding rewarded or non-rewarded outcomes, we analyzed each choice as a function of the outcome of the previous trial. This analysis revealed a significant treatment × feedback sensitivity (win-stay/lose-shift) interaction (*F*_(1,15)_ = 8.48, *p* = 0.01). Simple main effect analyses comparing baseline versus test days further revealed that inhibition of BLA inputs to the NAc before choice did not affect win-stay behavior (*p* > 0.17), but did significantly enhance lose-shift behavior (*p* = 0.003; Fig. 2*B*, inset). Additional exploratory analyses further partitioned the lose-shift effect across the two probability blocks. This revealed that the increase in negative feedback sensitivity in this experiment occurred primarily during the 50% block (baseline proportion of trials = 0.23 ± 0.06; test days = 0.35 ± 0.07; *F*_(1,15)_ = 11.91, *p* < 0.004) but not in the 12.5% block (baseline = 0.68 ± 0.06; test days = 0.63 ± 0.04; *F*_(1,15)_ = 1.52, *p* > 0.24). Thus, the reduction in risky choice during the higher probability block appeared to be driven by an increased tendency to shift to the small/certain option after a non-rewarded risky choice.

With respect to other performance measures, inhibition before choices caused slight, but statistically significant increases in choice latencies (*F*_(1,15)_ = 4.90, *p* = 0.04; Table 1) and trial omissions (*F*_(1,15)_ = 5.51, *p* = 0.03; Table 1). Although the effect of BLA→NAc inhibition on choice latencies was statistically reliable, in actuality, this amounted to ∼60 ms increase in response times between baseline and test, suggesting that at best, this manipulation, caused only a minor reduction in the incentive salience of the levers. Interestingly, these effects did not appear to be due to a general psychomotor slowing, as rats actually displayed a slight increase in locomotor on test days (*F*_(1,15)_ = 3.82, *p* = 0.07; Table 1). Taken together, these data indicate that optogenetic inhibition of BLA inputs to the NAc before choices flattened the discounting curve and reduced selection of more preferred options. Specifically, rats chose less risky in the higher probability block when they normally displayed a choice bias toward the risky option and chose more risky in the lower probability block when they normally preferred the small/certain option that had greater utility.

**Table 1:** Performance measures after BLA→NAc optogenetic inhibition during discrete periods of probabilistic discounting

	Mean (SEM)			
Manipulation	Baseline		BLA→NAc inhibition	
Prior to choice				
Response latency (s)	0.51	(0.03)	0.60*	(0.05)
Number of omissions (over 60 trials)	0.40	(0.10)	0.76*	(0.16)
Locomotion	1366	(86)	1522	(109)
Risky loss				
Response latency (s)	0.47	(0.03)	0.48	(0.03)
Number of omissions (over 60 trials)	0.22	(0.09)	0.23	(0.14)
Locomotion	1487	(120)	1473	(119)
Risky win				
Response latency (s)	0.43	(0.02)	0.43	(0.03)
Number of omissions (over 60 trials)	0.11	(0.05)	0.25	(0.13)
Locomotion	1503	(163)	1560	(146)
Small win				
Response latency (s)	0.42	(0.03)	0.42	(0.04)
Number of omissions (over 60 trials)	0.32	(0.11)	0.18	(0.11)
Locomotion	1531	(145)	1545	(143)
ITI				
Response latency (s)	0.40	(0.05)	0.42	(0.07)
Number of omissions (over 60 trials)	0.19	(0.12)	0.33	(0.14)
Locomotion	1720	(190)	1716	(146)
	Baseline		eYFP-only rats	
Prior to choice				
Response latency (s)	0.62	(0.07)	0.56	(0.08)
Number of omissions (over 60 trials)	0.50	(0.16)	0.19	(0.13)
Locomotion	1290	(136)	1307	(134)
Risky loss				
Response latency (s)	0.55	(0.07)	0.57	(0.06)
Number of omissions (over 60 trials)	0.18	(0.14)	0.21	(0.10)
Locomotion	1559	(199)	1501	(235)

Values are displayed as mean (SEM); *p < 0.05 between baseline and pathway inhibition.

### Inhibition following risky losses

**Figure 3.**
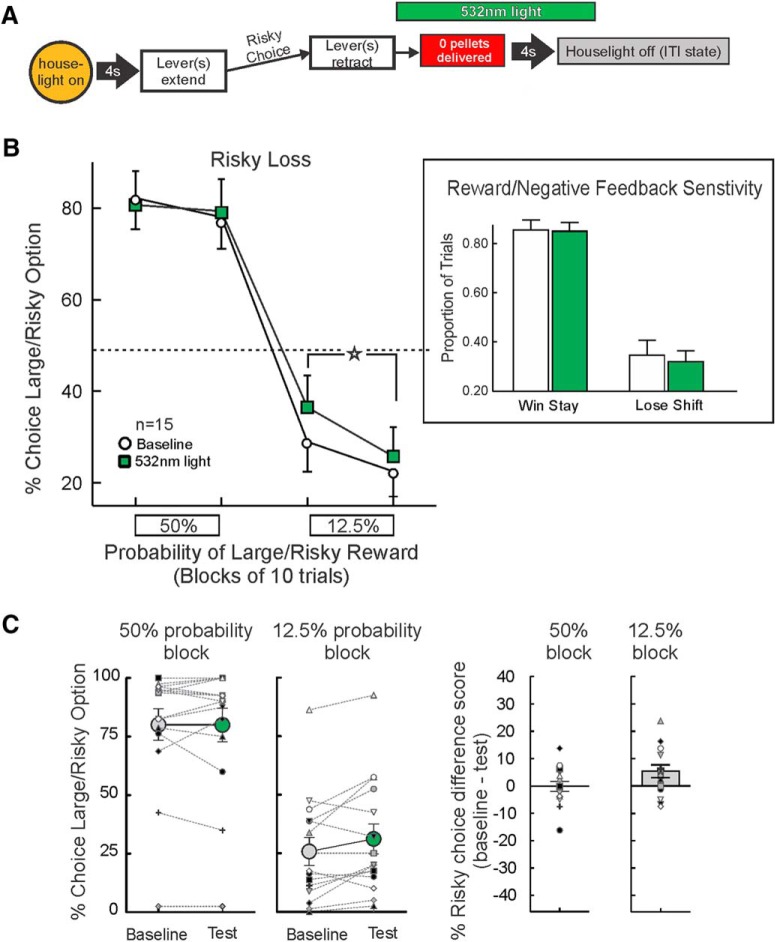
Inhibition of BLA→NAc pathway after risky losses increases risky choice. ***A***, Optogenetic manipulation and task design. Laser light was delivered for 7 s on trials when the large/risky lever was chosen and no reward was delivered. ***B***, Inhibition during risky losses increased choice of the large/risky option during the low probability block (*n* = 15). Inset, Win-stay/lose-shift data. The increase in risky choice was not associated with changes in win-stay/lose-shift behavior. ***C***, Individual data. In the majority of rats tested, optogenetic inhibition after risky losses increased risky choice relative to baseline in the 12.5% probability blocks.

The BLA→NAc pathway was optogenetically inhibited in 15 rats during a period coinciding with reward omissions following selection of the large/risky lever (Fig. 3*A*). Analysis of the choice data from this experiment did not yield a main effect of treatment (*F*_(1,14)_ = 2.27, *p* = 0.16), but did reveal a significant treatment × probability block interaction (*F*_(1,14)_ = 5.74, *p* = 0.03; Fig. 3*B*), but no three-way interaction (*F*_(1,14)_ = 1.69, *p >* 0.21). This effect was driven by an increase in choice of the large/risky lever during the low (12.5%) probability block compared to baseline (*F*_(1,14)_ = 11.13, *p* = 0.005), whereas there was no change in choice behavior during the 50% block (*p* > 0.50).

The individual choice data from this experiment is displayed in Figure 3*C*. In these 15 rats, inhibition of BLA→NAc inputs did not induce a reliable change in risky choice relative to baseline in the 50% probability block (six increased, eight decreased, two no change). However, in the 12.5% block, 10/15 rats chose the risky option more often on test days relative to their performance under baseline conditions.

Choice-by-choice analyses showed that this increase in risky choice was not driven by changes in win-stay or lose-shift behavior [main effect of treatment (*F*_(1,14)_ = 0.38, *p* > 0.50); treatment × response type interaction (*F*_(1,14)_ = 0.41, *p* > 0.50; Fig. 3*B*, inset)]. There were no significant effects of risky loss inhibition on locomotion, trial omissions or response latencies (all *F*s < 1.20, all *p*s > 0.30; Table 1). Thus, optogenetic inhibition of BLA→NAc inputs after non-rewarded actions increased bias for the risky option, with this effect emerging during the low probability block when the small/certain option was more advantageous.

### Inhibition following risky wins

**Figure 4.**
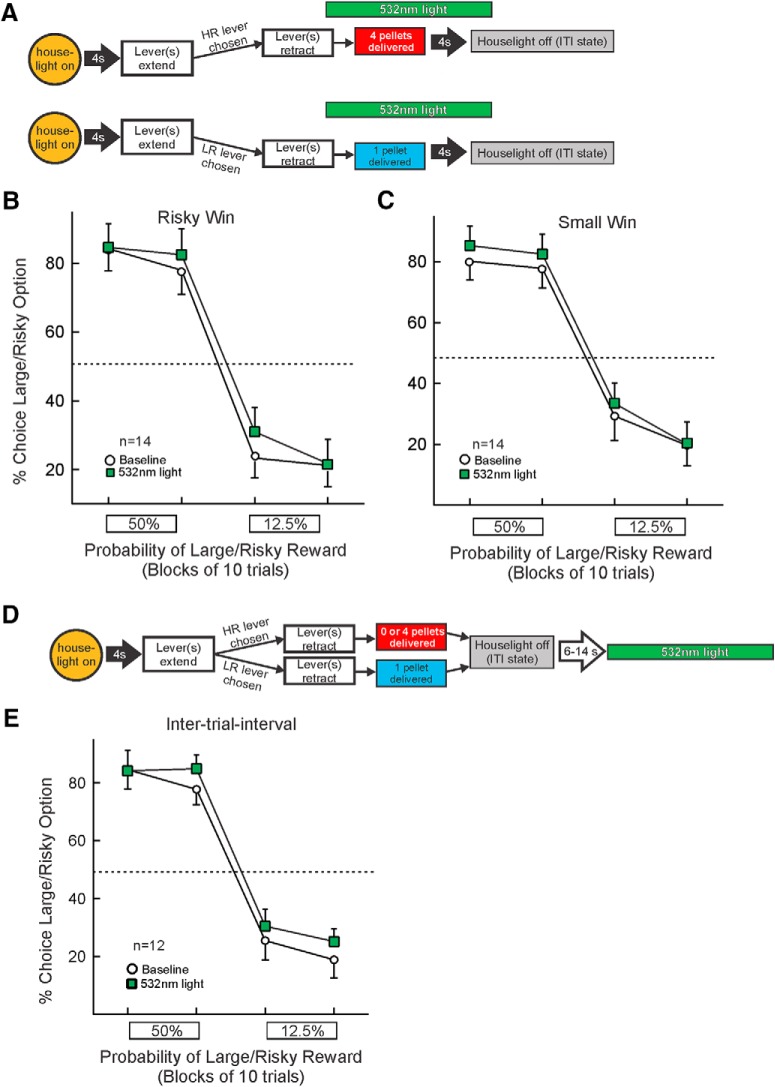
Inhibition of BLA→NAc pathway following rewarded outcomes and during ITI. ***A***, Optogenetic manipulation and task design. Top, Inhibition during large rewards occurred on trials the large/risky lever was chosen and reward was delivered. Bottom, Inhibition during small rewards occurred on trials immediately after the small/certain lever was chosen. ***B***, ***C***, Inhibition of pathway during either large/risky wins (*n* = 14) or small/safe wins (*n* = 14) shows no reliable change in choice. ***D***, Optogenetic manipulation during ITI. Light was delivered during every ITI, starting 6–12 s after the end of a trial. ***E***, Optogenetic inhibition during the ITI did not alter choice (*n* = 12).

In 14 rats, the BLA→NAc pathway was inhibited from the time rats chose the large/risky lever and received the large reward (Fig. 4*A*). As opposed to the effects of inhibiting after risky losses, inhibition following risky wins revealed no significant changes in choice (main effect of treatment: *F*_(1,13)_ = 1.67, *p* > 0.20; treatment × block interaction: *F*_(1,13)_ = 0.25, *p* > 0.60; three-way interaction: *F*_(1,13)_ = 4.65, *p* = 0.05; Fig. 4*B*). Furthermore, there were no significant effects of inhibition after risky wins on locomotion, trial omissions or response latencies (all *F* values < 1.78, all *p*s > 0.20; Table 1). Thus, silencing neural activity from BLA inputs to the NAc after obtaining larger, risky rewards did not affect subsequent choice behavior.

### Inhibition following small/certain wins

Similar to the lack of effect of silencing after risky wins, optogenetic inhibition of BLA→NAc inputs after rats chose the small/certain lever and received the smaller reward (*n* = 14) did not affect choice (main effect of treatment: *F*_(1,13)_ = 3.50, *p* = 0.08; treatment × probability block interaction: *F*_(1,13)_ = 0.96, *p* > 0.35; three-way interaction: *F*_(1,13)_ = 0.39, *p >* 0.54; Fig. 4*A*,*C*). As well, no significant changes in the other performance measures were apparent (all *F* values < 1.67, all *p*s > 0.20; Table 1). Ergo, inhibiting this pathway during receipt of small/certain rewards does alter subsequent action selection.

### Inhibition during the ITI

To establish the temporal specificity of outcome related effects associated with risky losses, 14 rats received optogentic inhibition of the BLA→NAc pathway during the ITI, starting at a randomized time point within each ITI (Fig. 4*D*). Visual inspection of Figure 4*E* suggest there appears to be a small increase in overall risky choice. However, in probing individual choice data revealed that this effect was driven primarily by 3 rats that showed an increase in risky choice. All other subjects either displayed no change or a decrease in risky choice. As such, the overall analysis of these data failed to yield a reliable significant effect (main effect of treatment (*F*_(1,13)_ = 0.83, *p* > 0.38; treatment × block interaction (*F*_(1,13)_ = 0.11, *p* > 0.74); three-way interaction; *F*_(1,13)_ = 0.49, *p* > 0.49). Furthermore, there were no significant effects for the other performance measures (all *F* values < 1.45, all *p*s > 0.25; Table 1). These findings indicate that the effects of BLA→NAc inhibition after risky losses were dependent on the temporal specificity of suppressing BLA terminal activity coincidental to when the outcomes of the preceding choices were realized.

### Control eYFP-only animals

**Figure 5.**
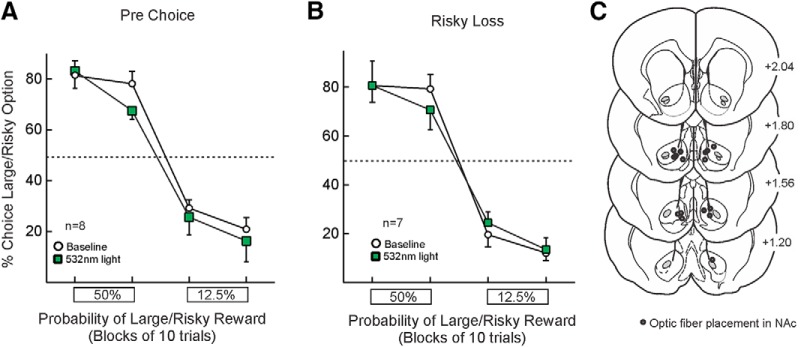
Laser light delivery in control, eYFP-only animals. Laser delivery into NAc (***A***) during the period before free-choice trials (*n* = 8) or (***B***) coinciding with non-rewarded choices (*n* = 7) did not affect choice. ***C***, Fiber optic placements in the NAc for eYFP-only animals.

In a separate cohort of rats infused with a virus that only encoded for eYFP, delivery of laser light did not affect choice when administered before choice (*n =* 8 rats; main effect of treatment (*F*_(1,7)_ = 1.21, *p* = 0.31); treatment × block interaction (*F*_(1,11)_ = 0.002, *p* = 0.96). Similarly, inhibition after risky losses also did not alter choice relative to baseline (*n =* 7 rats; main effect of treatment (*F*_(1,11)_ = 0.12, *p* = 0.74); treatment × block interaction (*F*_(1,11)_ = 1.13, *p* = 0.33; Fig. 5*A*,*B*). Location of fiber tips in these animals are presented in Figure 5*C*. These findings confirm that the effects on choice observed in rats treated with the active virus were not attributable to non-specific alterations induced by light application.

### Neurophysiological confirmation of BLA→NAc inhibition

**Figure 6.**
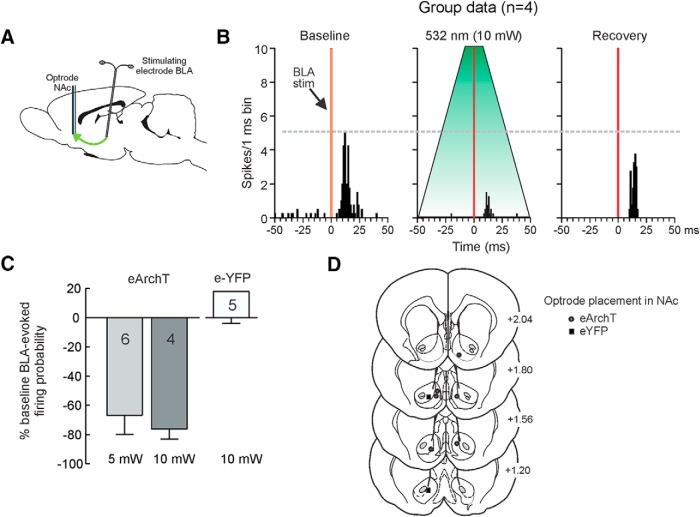
Neurophysiological confirmation of optogenetic inhibition of BLA→NAc inputs. ***A***, Experimental design. Stimulating electrodes were implanted in the BLA and optrode in NAc to record evoked firing of NAc neurons. ***B***, Peristimulus time histograms representing averaged evoked firing rates in 1-ms bins for four neurons (group data) during baseline sweeps (left), sweeps where 10-mW light was applied around the time of BLA stimulation (middle) and recovery (right). Application of light markedly suppressed evoked firing. ***C***, Average percentage change from baseline in BLA-evoked firing probability for rats infused with eArchT in the BLA that received 5- and 10-mW light application, and from control rats infused with eYFP. ***D***, Optrode placements in the NAc. Symbols represent location of last cell recorded from each rat.

To confirm that our optogenetic manipulations could suppress neural activity of in the BLA→NAc pathway, another experiment used *in vivo* single-unit recordings of NAc neurons to confirm that 532-nm light application within the NAc could attenuate firing of neurons driven by the BLA (Fig. 6*A*). In one group of cells (*n* = 4) obtained from two rats that received intra-BLA infusions of a virus encoding for eArchT, application of 10-mW light pulses for 4 s around the time of BLA stimulation caused a 76 ± 7% reduction in BLA-evoked firing compared to baseline (firing probabilities: baseline = 59 ± 11%; laser = 15 ± 7%; *F*_(1,3)_ = 35.00, *p* = 0.001; Fig. 6*B*,*C*). Notably, when we administered another series of stimulation pules to the BLA ∼2 min after those that coincided with light application, evoked firing recovered to baseline, and we did not see any increase in spontaneous or evoked activity (Fig. 6*B*, recovery). Similarly, in another group of cells (*n* = 6) recorded from four rats infused with eArchT, application of 5-mW light during BLA stimulation caused a 67 ± 14% reduction in evoked firing of NAc neurons relative to baseline (firing probabilities: baseline = 53 ± 13%; laser = 25 ± 12%; *F*_(1,5)_ = 9.92, *p =* 0.03; Fig. 6*C*). In contrast, data obtained from five cells recorded from two rats infused with virus that encoded eYFP, but not the inhibitory opsin, application of light around the time of BLA stimulation did not reliably alter evoked firing (firing probabilities; baseline = 62 ± 13%; laser = 62 ± 9%; *F*_(1,4)_ = 0.002, *p* > 0.90; Fig. 6*C*). These data confirm that application of 532-nm light in the NAc can suppress firing of NAc neurons driven by inputs from the BLA, but only in rats receiving intra-BLA infusions of the virus promoting expression of eArchT.

## Discussion

It is well established that BLA→NAc circuitry plays an integral role in facilitating a variety of reward-related behaviors. Studies using conventional lesion/inactivation and disconnection procedures have implicated this pathway in mediating approach to reward-associated stimuli (Everitt et al., 1991; Ambroggi et al., 2008; Chang et al., 2012), sensitivity to outcome devaluation (Shiflett and Balleine, 2010), extinction of instrumental reward seeking and subsequent expression of extinction learning (McLaughlin and Floresco, 2007; Millan and McNally, 2011), probabilistic reinforcement learning (Averbeck and Costa, 2017), and of particular relevance, probabilistic discounting (St Onge et al., 2012). Here, we exploited the temporal precision afforded by optogenetic inhibition to elucidate how activity in this pathway, during different phases of the decision process, influences action selection during risk/reward decision making. Inhibition before choice reduced the tendency for rats to select higher-utility options they normally preferred under baseline conditions. In comparison, attenuating activity in this circuit after non-rewarded risky choices increased bias toward large/risky rewards when the probability of obtaining them were low. Similar manipulations after rewarded choices or during the ITI were without effect. These findings highlight how activity in the BLA→NAc pathway either before action selection or after their outcomes are realized can differentially influence choice between rewards of different magnitudes and probabilities.

### BLA→NAc activity before action selection promotes choice of higher-value options

Inhibition of BLA→NAc inputs when rats were deliberating about their choices shifted choice biases away from their preferred option. When reward probabilities were comparatively high (50%) choice was biased toward the risky option during baseline, yet pre-choice inhibition of BLA inputs to the NAc reduced preference of this option. This was associated with enhanced sensitivity to reward omissions (i.e., increased lose-shift behavior), similar to that observed after pre-choice silencing of the BLA in rats choosing between larger/punished rewards and smaller/safe ones (Orsini et al., 2017). The present findings suggest signals from the BLA to the NAc serve to dampen the aversive impact of recent non-rewarded actions and promote choice of larger, higher-probability rewards. In contrast, when reward probabilities were low (12.5%), this manipulation increased risky choice, shifting bias away from the more-preferred small/certain option. Thus, these manipulations did not induce a uniform increase or decrease in risky choice, but instead, led to sub-optimal patterns of decision making. It is unlikely that these effects are attributable to non-specific disruptions in discriminating between larger versus smaller rewards or the spatial position of levers, because BLA→NAc disconnections do not affect preference of larger versus smaller rewards when both are delivered with 100% probability (St Onge et al., 2012). Rather, the BLA may be part of a broader circuit (including prefrontal cortices; St Onge and Floresco, 2010; Jenni et al., 2017) that computes relative values of rewards that differ in terms of the uncertainty and magnitude. During periods when a decision maker is deliberating, signals from BLA to the NAc appear to promote approach toward targets of greater subjective value.

These effects of pre-choice suppression of BLA→NAc activity on risk/reward decision making complements previous neurophysiological studies describing choice-related activity of NAc neurons during risk/reward decision making. Sugam et al. (2014) showed that when rats were choosing between larger/uncertain and smaller/certain rewards, NAc neurons displayed phasic changes in activity during periods before a choice was made. Importantly, these changes were more robust when rats chose their more preferred option, compared to the activity when they ultimately chose a less-preferred option. These findings, juxtaposed to the present results, make it reasonable to propose that neural encoding of choice preferences in NAc neurons is driven in part by inputs from the BLA, and that this activity increases the likelihood that actions are directed toward more preferred rewards.

The ability of BLA→NAc signals to promote selection of reward of greater subjective value may be facilitated by dopaminergic modulation of activity in this pathway. Activation of the BLA can promote mesoaccumbens dopamine release via local, glutamate-receptor dependent mechanisms (Floresco et al., 1998; Howland et al., 2002; Jones et al., 2010). In turn, dopamine released by this mechanism can facilitate BLA-evoked firing of NAc neurons via actions on the D_1_ receptor (Floresco et al., 2001). In this regard, blockade of NAc D_1_ receptors reduces choice of larger/risky rewards and increases lose-shift behavior (Stopper et al., 2013), similar to what was observed in the present study. Taken together, it is plausible that during periods leading up to choice initiation, converging BLA and dopaminergic inputs (acting on D_1_ receptors) may work cooperatively to enhance phasic firing of NAc neurons that in turn increases the likelihood that actions are directed toward more preferred options.

### BLA→NAc communication after non-rewarded actions modifies choice

In another experiment, suppressing activity in the BLA→NAc pathway immediately after a non-rewarded action increased risky choice during the low probability block. These findings complement the observation that reward omissions after a risky choice increase phasic firing in a subpopulation of NAc neurons (Sugam et al., 2014), and suggest that this outcome-related activity may be driven in part by input from the BLA (Roesch et al., 2010). Interestingly, this effect was not associated with an increased tendency to shift to the small/certain option on trials following a loss. Instead, inhibiting activity over multiple reward omissions appeared to diminish the aversive impact of repeated losses experienced over the session, rather than immediately after a loss. This suggest that repeated bursts of activity in this pathway after non-rewarded actions may serve to accumulate information about losses over time (potentially mediated by short-term plasticity within this circuitry) that in turn modifies decision biases over a longer period.

Classical views of amygdala function posit that this region mediates responding to aversive stimuli such as punishment or reward omissions (Balleine and Killcross, 2006; Winstanley and Floresco, 2016). The degree to which animals perceive reward omissions as being aversive may influence the degree to which they attempt to avoid these losses (Baxter and Murray, 2002). With respect to reward-related decision making, the BLA has been proposed to assign value to a reward option, and in using accumulated information about the absence of reward from multiple trials to guide subsequent behavior (Cardinal et al., 2002; Averbeck and Costa, 2017). The present data indicate that in addition to biasing choice before action selection, signals from the BLA linked to non-rewarded actions are relayed to the NAc, and this activity can gradually shape choice bias away from options that are rewarded less frequently.

In contrast to the above-mentioned findings, inhibition of BLA→NAc activity during receipt of larger or smaller rewards did not alter choice. This lack of effect was interesting, considering both BLA and NAc neurons increase firing to rewards and encode differences in reward magnitude (Lavoie and Mizumori, 1994; Pratt and Mizumori, 1998; Belova et al., 2008; Roesch et al., 2010; Sugam et al., 2014). Notably, Orsini et al. (2017) also observed that optogenetic inhibition of the BLA after rewarded actions did not affect choice in rats selecting between smaller rewards and larger ones associated with varying probabilities of punishment. Thus, although rewards can increase firing of both BLA and NAc neurons, our data suggest that rewarded outcome-related activity in this circuitry does not play a major role in biasing subsequent choice. Instead, reward-associated phasic activity within the mesoaccumbens dopamine pathway may play a more prominent role in influencing subsequent choice biases. Reward delivery during risk/reward decision making is associated with phasic increases in NAc dopamine release, with larger/risky rewards evoking greater responses compared to smaller, certain ones (Sugam et al., 2012). Furthermore, it has been shown that temporally-discrete suppression of phasic dopamine activity (via stimulation of the lateral habenula) during delivery of larger/risky or small/certain rewards reduces or increases risky choice, respectively (Stopper et al., 2014). Conversely, non-rewarded risky choices induce a brief reduction or “dip” in dopamine activity (Sugam et al., 2012), and stimulation of the midbrain dopamine neuron region after non-rewarded actions increased risky choice (Stopper et al., 2014). These data suggest that outcome-related phasic bursts or dips in dopamine activity provide information about recent rewarded or non-rewarded actions that can influence subsequent choices. Integration of these findings with the present data provides insight into the dynamic interplay of different outcome-related signals that work in concert at the level of the NAc to refine ongoing reward seeking when reward probabilities are volatile. Phasic increases in NAc dopamine linked to rewarded choices serve to increase the likelihood that these actions are repeated. In comparison, after non-rewarded choices, phasic activation of glutamatergic BLA inputs, combined with brief suppression of dopaminergic activity may play a complementary role that promotes exploration of other options.

### Neurophysiological considerations

In a separate experiment, we confirmed that light application markedly attenuated BLA-evoked firing of NAc neurons, but only in rats that expressed the inhibitory opsin. Notably, this did not induce a complete “silencing” of evoked firing, which may explain why the magnitude of our behavioral effects were somewhat smaller compared to those induced by conventional inactivations (St Onge et al., 2012). Nevertheless, the fact that we could cause a significant and reliable suppression in evoked neural firing highlights that optogenetic inhibition can be used to degrade signal integrity of terminal inputs to a brain region during different task events, that in turn is sufficient to alter complex behavior.

## Conclusions

Collectively, the present data provide novel insight into the temporal dynamics of activity in BLA→NAc circuitry during deliberative and evaluative components of cost/benefit decision making and how this may guide action selection in situations involving reward uncertainty. These findings complement studies on the neural basis of decision making in humans, implicating the amygdala and NAc in guiding neuroeconomic choices and appraisals of their outcomes (Matthews et al., 2004; Kuhnen and Knutson, 2005; Knutson et al., 2008; Gelskov et al., 2015). Moreover, abnormal patterns of activation in the BLA and NAc have been observed in a variety of populations that display suboptimal or maladaptive patterns of decision making, including individuals with obsessive/compulsive disorder (Admon et al., 2012), anxious youth (Galván and Peris, 2014), and healthy adolescents versus adults (Ernst et al., 2005). Clarifying how dynamic patterns of activity within different nodes of cortico-limbic-striatal circuitry during different phases of the decision process guide reward seeking will ultimately aid in understanding the neural mechanisms that underlie optimal and aberrant patterns of decision making.

## Synthesis

Reviewing Editor: Mark Baxter, Mount Sinai School of Medicine Decisions are customarily a result of the Reviewing Editor and the peer reviewers coming together and discussing their recommendations until a consensus is reached. When revisions are invited, a fact-based synthesis statement explaining their decision and outlining what is needed to prepare a revision will be listed below. Note: If this manuscript was transferred from JNeurosci and a decision was made to accept the manuscript without peer review, a brief statement to this effect will instead be what is listed below.

“The manuscript was accepted after editorial review.”
